# Multipotent Neural Crest Stem Cell-Like Cells from Rat Vibrissa Dermal Papilla Induce Neuronal Differentiation of PC12 Cells

**DOI:** 10.1155/2014/186239

**Published:** 2014-06-18

**Authors:** Meiying Li, Jin Yu Liu, Shichao Wang, Hao Xu, Lifeng Cui, Shuang Lv, Jinying Xu, Shutong Liu, Guangfan Chi, Yulin Li

**Affiliations:** ^1^The Key Laboratory of Pathobiology, Ministry of Education, Jilin University, 126 Xinmin Street, Changchun, Jilin 130021, China; ^2^Department of Gynaecology, Third Hospital of Jilin University, Changchun, China; ^3^Department of Biological Engineering, College of Pharmacy, Jilin University, Changchun, China; ^4^Department of Gynaecology, First Hospital of Jilin University, Changchun, China

## Abstract

Bone marrow mesenchymal stem cells (BMSCs) transplants have been approved for treating central nervous system (CNS) injuries and diseases; however, their clinical applications are limited. Here, we model the therapeutic potential of dermal papilla cells (DPCs) *in vitro*. DPCs were isolated from rat vibrissae and characterized by immunocytofluorescence, RT-PCR, and multidifferentiation assays. We examined whether these cells could secrete neurotrophic factors (NTFs) by using cocultures of rat pheochromocytoma cells (PC12) with conditioned medium and ELISA assay. DPCs expressed Sox10, P75, Nestin, Sox9, and differentiated into adipocytes, osteoblasts, smooth muscle cells, and neurons under specific inducing conditions. The DPC-conditioned medium (DPC-CM) induced neuronal differentiation of PC12 cells and promoted neurite outgrowth. Results of ELISA assay showed that compared to BMSCs, DPCs secreted more brain-derived neurotrophic factor (BDNF) and glial cell line-derived neurotrophic factor (GDNF). Moreover, we observed that, compared with the total DPC population, sphere-forming DPCs expressed higher levels of Nestin and P75 and secreted greater amounts of GDNF. The DPCs from craniofacial hair follicle papilla may be a new and promising source for treating CNS injuries and diseases.

## 1. Introduction

Unlike most tissues, central nervous system (CNS) tissue has a limited capacity for self-repair because mature neurons lack the ability to regenerate. Stem cell-based therapies, however, have introduced new possibilities for repair and restoration of neuronal function after CNS injury [1, 2]. Among them, autologous adult stem cells such as bone marrow mesenchymal stem cells (BMSCs) have received more attention in recent years [3, 4].

Transplantation of BMSCs is a promising therapy for stroke, and some studies have reached the stage of clinical investigation [5, 6]. According to these reports, the underlying mechanisms of functional recovery following BMSCs transplantation are likely mediated by the release of neurotrophic factors (NTFs) and growth factors (including BDNF and GDNF). These factors likely promote endogenous repair mechanisms, reduce the occurrence of cell death, and stimulate neurogenesis and angiogenesis, rather than promote neuronal differentiation or implant integration at the ischemic site [7–9]. Nevertheless, BMSCs must be isolated by bone marrow aspiration, which is traumatic and painful. Moreover, the percentage of stem cells in bone marrow is very low (0.001–0.01%) and decreases with age, thus making it difficult to harvest a sufficient number of high-quality cells for clinical application [10]. For these reasons, there is a need to find other autologous sources of adult stem cells.

The neural crest, which is limited to vertebrates, is a versatile embryonic tissue. When grown in specified medium, neural crest cells can differentiate into a number of neural crest derivatives including neurons, glia, melanocytes, smooth muscle cells (SMCs), chondrocytes, and osteoblasts [11, 12]. Therefore, these neural crest cells have been termed “neural crest stem cells” (NCSCs). Several adult tissues retain NCSCs-like cells, including those from the dorsal root ganglia, bone marrow, gut, heart, cornea, carotid body, and skin [13, 14]. The papillae of rodent vibrissa follicles also originate from the neural crest. These tissues are enriched with precursor cells that have both neuronal and glial potential, and that demonstrate therapeutic efficacy after transplantation into the injured spinal cord [15, 16]. To our knowledge, the underlying mechanisms of these therapeutic functions have not been investigated. We postulated that multipotent stem cells derived from craniofacial hair follicle papilla may be a promising cell source for treating CNS injury. The aim of this study was to investigate the properties of dermal papilla cells (DPCs) isolated from rat vibrissa follicles, and to compare them with BMSCs from the same rat. Specifically, we examined their multidifferentiation potential and secretion of NTFs* in vitro*.

## 2. Materials and Methods

### 2.1. Animals

Adult male Wistar rats (200–250 g, *n* = 7) were supplied by the Experimental Animal Center of Jilin University. All experimental procedures were approved by the ethics committee of Jilin University and conformed to the regulatory standards.

### 2.2. Isolation of Vibrissa Dermal Papillae and Cultivation of DPCs

The rats were deeply anesthetized with an intraperitoneal injection of 10% chloral hydrate and were killed by cervical dislocation. The whisker pads were cut free from surrounding tissue under aseptic conditions and thoroughly washed with phosphate buffered saline (PBS) containing 5% (*v*/*v*) penicillin and streptomycin (HyClone, Logan, UT). The inner surface of the whisker pad was exposed, and the vibrissa follicles were dissected using small scissors under a stereoscopic microscope. The isolated follicles were then stored in sterile culture medium for further analysis. The subcutaneous fat and connective tissue surrounding the collagen capsules were then carefully removed. Using microscissors, we made a transverse incision at the top part of the collagen capsule and then a longitudinal incision on the remaining part of the capsule that extended downward to just above the hair bulb. Afterward, the hair shaft was held with microforceps and the follicle structure was carefully pulled away from collagen capsule. During this operation, special care was taken to keep the structure of the bulb intact. The separated follicles were digested with 0.1% collagenase type I (Sigma, St. Louis, USA) for 1 h at 37°C. Subsequently, the papillae were dissociated by gentle pipetting and transferred to another culture dish using microforceps. Finally, the papillae were cultured in DMEM/F-12 (1 : 1) medium (Gibco, Paisley, UK) supplemented with 10% (*v*/*v*) fetal bovine serum (FBS, HyClone) and 10 ng/mL basic fibroblast growth factor (bFGF; PeproTech, London, UK), which hereafter is referred to as the proliferation medium. Half of the medium was changed every 3 days. The DPCs that migrated out of the explants were digested with 0.25% trypsin (Invitrogen, Carlsbad, CA) and 0.02% EDTA (Sigma), and then expanded in new culture dishes at 1 × 10^4^ cells/cm^2^. The cells were passaged when they reached ~90% confluence. As a control, BMSCs were isolated from the same donor rat using the method described in the Supplementary Material available online at http://dx.doi.org/10.1155/2014/186239.

### 2.3. Alkaline Phosphatase (ALP) Staining

The expression of ALP was detected using a BCIP/NBT ALP color development kit (Bi Yuntian, Shanghai, China) according to the manufacturer's protocol.

### 2.4. Reverse Transcriptase-Polymerase Chain Reaction (RT-PCR) Analysis

Total RNA was extracted using Trizol reagent (Invitrogen) according to the manufacturer's protocol. cDNA was synthesized from 500 ng of total RNA using the Takara RNA polymerase chain reaction (PCR) kit (AMV) version 3.0 (Takara, Dalian, China). The PCR was carried out with 1 *μ*L cDNA in a 20 *μ*L reaction volume using a PCR kit (Kang Wei Shi Ji, Beijing, China). The PCR product was detected using 1.5% agarose gel electrophoresis. Detailed information on these primers is listed in Table 1.

### 2.5. Immunocytofluorescence Staining

The DPCs were fixed in 4% paraformaldehyde (Ding Guo, Beijing, China) for 10 min and permeabilized with 0.1% Triton X-100 (Sigma) for 20 min at room temperature. Normal goat serum (10%; Maixin, Fuzhou, China) was used to block nonspecific binding. The DPCs were then incubated overnight with primary antibodies at 4°C (detailed information regarding the primary antibodies is listed in Table 2), followed by incubation with Alexa Fluor 488/555-conjugated secondary antibodies (Cell Signaling Technology, Danvers, USA) for 1 h at room temperature. Unbound antibody was removed by thoroughly washing with PBS. Nuclei were stained with Hoechst 33342 (Invitrogen). For the negative control, primary antibodies were omitted.

### 2.6. Flow Cytometry Analysis

The DPCs were trypsinized and counted. Approximately 1 × 10^6^ cells were used for each test; the cells were rinsed with PBS by centrifugation at 4°C, resuspended with 1% BSA/PBS, and incubated for 30 min. Next, the DPCs were incubated with primary antibodies for 1 h on ice (the detailed information for the primary antibodies is listed in Table 2), followed by incubation with Alexa Fluor 488-conjugated secondary antibodies for 1 h. The labeled cells were thoroughly washed with PBS and analyzed on a BD FACSCalibur flow cytometer (BD Biosciences, San Jose, CA). The primary antibodies were omitted as a negative control.

### 2.7. Multipotent Differentiation Assay

#### 2.7.1. Adipogenic Differentiation

Adipogenic differentiation was induced by culturing cells at 90% confluence in DMEM/F-12 (1 : 1) medium supplemented with 10% (*v*/*v*) FBS, 0.5 mM IBMX (Sigma), 0.2 mM Indocin (Sigma), 1 *μ*M dexamethasone (Sigma), and 10 mg/L insulin (Sigma) for 2 weeks. Half of the medium was changed every 3 days. Intracellular lipid droplets were detected using Oil-Red O staining as previously described [17].

#### 2.7.2. Osteogenic Differentiation

Osteogenic differentiation was induced by culturing cells at 90% confluence in DMEM/F-12 (1 : 1) medium supplemented with 10% (*v*/*v*) FBS, 0.1 *μ*M dexamethasone, 50 *μ*g/mL l-ascorbic acid 2-phosphate (Sigma), and 10 mM *β*-glycerophosphate (Sigma) for 2 weeks. Half of the medium was changed every 3 days. Mineralized bone nodules were detected using Alizarin Red-S [18].

#### 2.7.3. SMC Differentiation

SMC differentiation was induced by culturing cells at 50% confluence in DMEM/F-12 (1 : 1) medium supplemented with 1% (*v*/*v*) FBS for 1 week. The differentiated cells were evaluated by immunofluorescence staining for the expression of *α*-SMA. For quantification analysis, images of 6 fields per well were randomly selected by fluorescence microscopy (Olympus, Tokyo, Japan) at 400× magnification. The differentiation efficiency was expressed as the ratio of *α*-SMA-positive cells to the total number of cells labeled with Hoechst 33342 [19].

#### 2.7.4. Neuronal Differentiation

Neuronal differentiation was induced by culturing cells at 90% confluence in DMEM/F-12 (1 : 1) medium supplemented with 2% (*v*/*v*) FBS, 100 ng/mL bFGF, 10 *μ*M forskolin (Sigma) for 2 days, followed by culturing in DMEM/F-12 (1 : 1) medium supplemented with 2% FBS, 50 ng/mL BDNF (PeproTech), 50 ng/mL nerve growth factor (NGF; R&D Systems, Wiesbaden, Germany), 10 ng/mL NT3 (PeproTech), 5 *μ*M forskolin, and 2% (*v*/*v*) B-27 (Gibco) for 5 days. The differentiated cells were evaluated with immunofluorescence staining for the expression of neuron-specific *β*3-Tubulin. For quantification, images of 6 fields per well were randomly selected by fluorescence microscopy at 200× magnification and the differentiation efficiency was expressed as the ratio of *β*3-Tubulin-positive cells with neuron-like morphology to the total number of cells labeled with Hoechst 33342.

### 2.8. Assessment of NTFs Secretion and Bioactivity

On the fourth passage, the DPCs and BMSCs were seeded in T25 flasks at a density of 1 × 10^4^/cm^2^, and were thoroughly washed with PBS when they had reached ~95% confluence. Next, the cells were cultured in 5 mL of fresh basal medium (BM) consisting of DMEM/F-12 (1 : 1) medium supplemented with 5% (*v*/*v*) FBS and 5% (*v*/*v*) equine serum (HyClone). After a 24 h cultivation, the supernatant was collected as DPC-conditioned medium (DPC-CM) and BMSC-conditioned medium (BMSC-CM), which were filtered through 0.22 *μ*m filters and frozen immediately at −20°C. The cells were trypsinized and counted.

Rat pheochromocytoma cells (PC12), which have been widely used to investigate the molecular mechanisms underlying neuronal differentiation [20], were purchased from ATCC (Rockville, MD, USA). For the bioactivity analysis, PC12 cells were seeded in 24-well plates that were previously coated with poly-l-lysine (Sigma) at a density of 5 × 10^3^ cells/well, and were cultured with BM overnight in a 37°C/5% CO_2_ incubator. On the following day, media in each well was switched to one of the experimental conditions: BM, DPC-CM, or BM supplemented with 50 ng/mL NGF for 15 days. Half of the medium was changed every 2 days.

To determine whether these media affected neurite growth, the neurite lengths of PC12 cells cultured in BM were compared with the lengths of those cultured in DPC-CM supplemented with various concentrations of NGF (0, 5, 10, and 30 ng/mL). Half of the medium was changed every 3 days. After culturing for 4 days, images were randomly captured by phase contrast microscopy at 400× magnification. The neurite length was quantified using Image-Pro Plus 6.0, and only neurites with lengths that were twice the cell body diameter were measured. The neuronal differentiation of PC12 cells was evaluated with immunofluorescence staining to identify expression of the neuron markers *β*3-Tubulin and NF-200KD [21].

To evaluate the NTFs, the concentrations of NGF, BDNF, and GDNF in DPC-CM, as well as BMSC-CM, were measured with ELISA kits (NGF, BDNF: Millipore Corp., California, USA; GDNF: Abnova, Taipei, Taiwan) according to the manufacturers' protocols. DPC-CM and BMSC-CM without dilution were added to 96-well immunoassay plates, at 100 *μ*L per well. Concentrations of these NTFs in BM were measured as the control. All samples were analyzed in triplicate. Finally, the secretion levels of NTFs were expressed as the total amount of each NTF secreted by 10 thousand cells in 5 mL conditioned medium for 24 h.

### 2.9. Isolation and Characterization of NCSCs-Like Cells

The DPCs were seeded in 6-well plates with an ultralow attachment at a density of 1 × 10^4^ cells/mL in 3 mL of serum-free sphere-forming medium, which consisted of DMEM/F-12 medium (1 : 1), 20 ng/mL bFGF, 20 ng/mL epidermal growth factor (EGF, PeproTech), 1% (*v*/*v*) N-2 (Gibco), and 2% (*v*/*v*) B-27. Two-thirds of the medium was changed every 4 days. After culturing for 7 days, sphere numbers were counted under low-power microscope, and the sphere-forming efficiency was expressed as a ratio of spheres to the initial number of single cells. Spheres and non-sphere-forming DPCs were separated by filtering through a 40 *μ*m mesh, followed by adherent culture in proliferation medium. For measuring the secretion of NTFs with ELISA, the sphere-forming DPCs and total DPCs at the same passage were thoroughly washed with PBS when they had reached ~95% confluence. They were then cultured in 5 mL of fresh proliferation medium, and after culturing for 24 h, the supernatants were collected and filtered through 0.22 *μ*m filters and immediately frozen at −20°C. The cells were trypsinized and counted.

### 2.10. Statistical Analysis

Statistical analysis was performed using SPSS software version 17.0. Data are presented as the mean ± SEM from at least three independent experiments. Multiple group comparisons were made using one-way analysis of variance (ANOVA). Two groups were tested by Student's *t*-test. Differences between groups were considered statistically significant at *P* < 0.05.

## 3. Results

### 3.1. Isolation of Rat Vibrissa Dermal Papillae and Cultivation of DPCs

**Figure 1:**
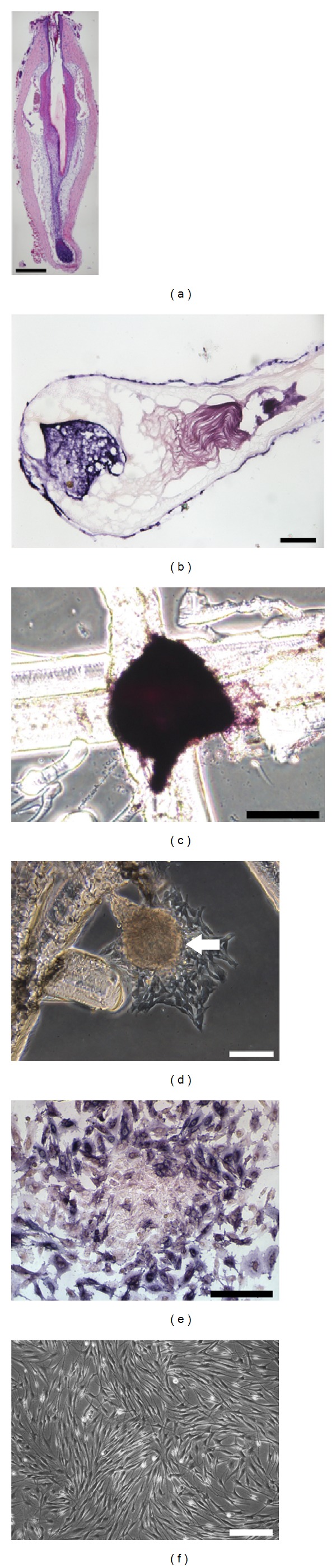
Isolation of rat vibrissa dermal papillae and cultivation of DPCs. (a) H&E staining of rat vibrissa hair follicle paraffin sections revealed the anatomic characteristics of dermal papilla. (b) Immunohistochemical staining of frozen sections revealed the expression of ALP in the dermal papilla and outer root sheath. (c) ALP was expressed in the isolated dermal papilla. (d) Spindle-shaped fibrocyte-like cells grew out from the “water-drop” shaped dermal papilla (white arrow). (e) ALP was partly expressed in primary DPCs. (f) Morphology of DPCs at passage 7. Scale bar = 100 *μ*m ((a), (b), (d)), 50 *μ*m ((c), (e)), and 200 *μ*m (f).

H&E staining of paraffin sections of rat vibrissa follicles revealed that the papillae were located inside the hair bulb and had a water-drop shape (Figure 1(a)). According to the immunohistochemical staining, ALP was expressed in the dermal papilla and outer root sheath; the positive reaction was identified by precipitation of blue-violet (Figure 1(b)). As shown in Figure 1(c), the isolated papilla-like structure expressed ALP, which indicated that we had successfully dissociated the papilla from the rat vibrissa follicle. In order to prevent the papilla from floating in the media, we made a small cross-shaped scratch on the bottom of the culture dish with microforceps under a stereoscopic microscope. We then gently placed a papilla on the scratched line. After these manipulations, all papillae were attached to the culture dish, and after 2 days of cultivation, the typical spindle-shaped fibrocyte-like cells grew from the explants (Figure 1(d)). The majority of the outward-migrating cells expressed ALP (Figure 1(e)). After seven passages, DPCs still showed prominent proliferative activity, and about 5 days later, they were arranged in a swirl that formed a confluent monolayer (Figure 1(f)). We simultaneously harvested bone marrow from the same donor rat and carried out primary culture of BMSCs (Supplementary Figure  1(a)). The expanded BMSCs showed a typical fibroblast-like morphology (Supplementary Figure  1(b)). Under our culture conditions, the proliferation rate declined with passage. After 4-5 passages, the cell proliferation ratio had obviously slowed, and the cells had changed to a flattened and spread out morphology (Supplementary Figure  1(c)).

### 3.2. Identification of Neural Crest Derived DPCs

**Figure 2:**
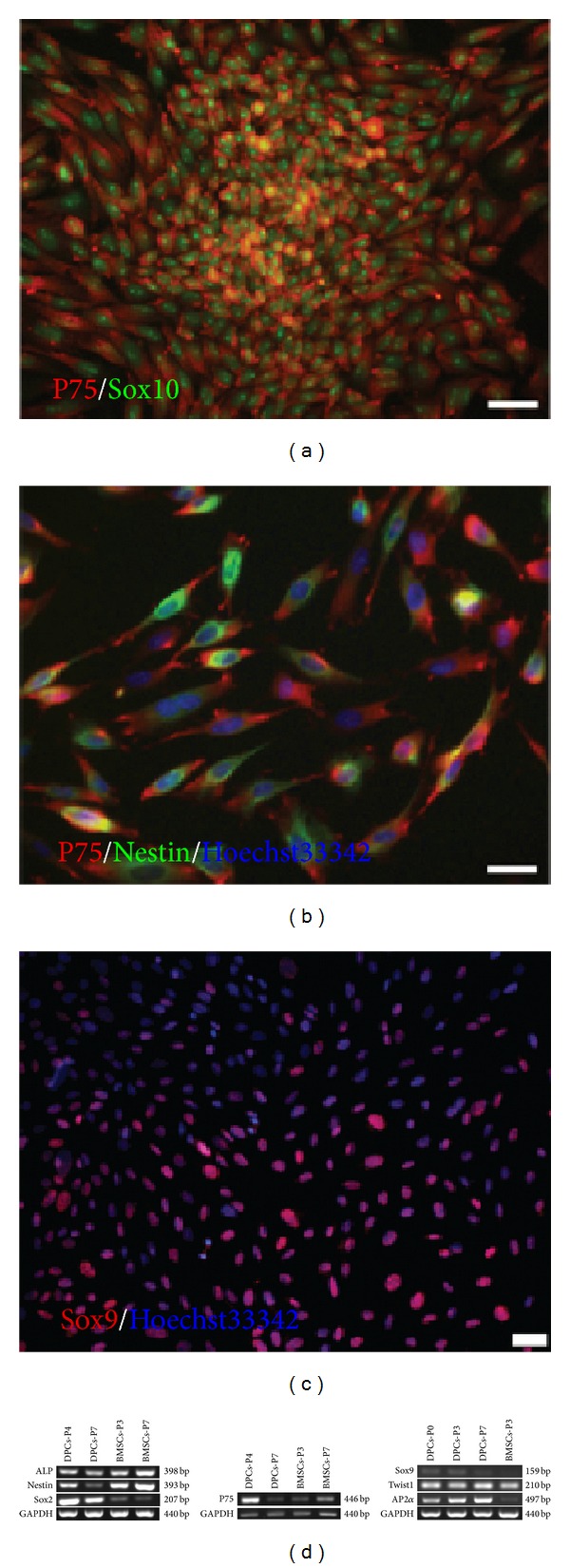
Identification of neural crest-derived DPCs. ((a)–(c)) Immunofluorescent cytochemical staining revealed expression of NCSCs-specific markers in DPCs. Primary DPCs were double positive for P75 and Sox10 (a), P75 and Nestin (b), and were positive for Sox9 (c). (d) RT-PCR analysis confirmed the expression of dermal papillae markers ALP and Sox2, and the NCSCs markers Nestin, P75, Sox9, Twist1, and AP2*α* in DPCs, as well as BMSCs. Scale bar = 20 *μ*m ((a)–(c)). DPCs-P#: DPCs at passage #; BMSCs-P#: BMSCs at passage #.

To investigate whether these DPCs expressed neural crest markers, we performed immunofluorescent staining. The primary DPCs were positive for P75, Sox10 (Figure 2(a)), Nestin (Figure 2(b)), and Sox9 (Figure 2(c)). After passage, DPCs were negative for Sox10 (Supplementary Figure  2(a)), but were still positive for P75 (Supplementary Figure  2(a)) and Nestin (Supplementary Figure  2(b)). RT-PCR analysis showed that DPCs strongly expressed the papillae markers ALP and Sox2, as well as NCSCs markers Nestin, P75, Sox9, Twist1, and AP2*α* (Figure 2(d)).

### 3.3. Analysis of Mesenchymal Stem Cell (MSC) Phenotype

**Figure 3:**
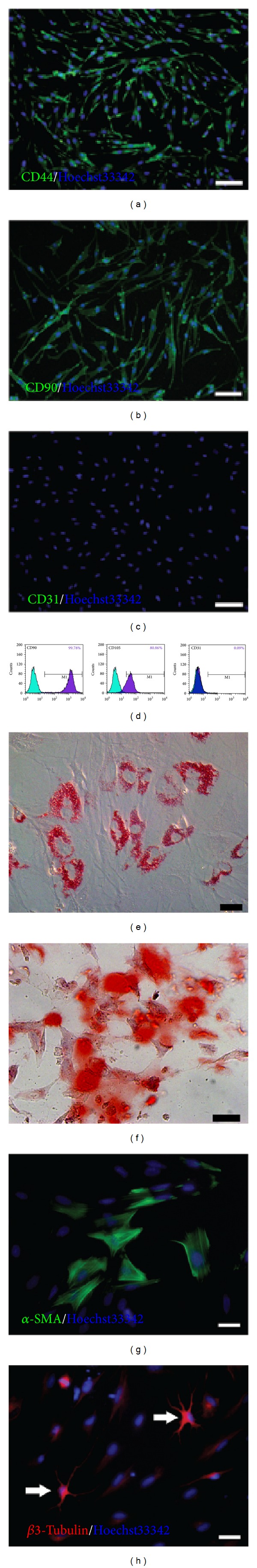
Mesenchymal stem cell phenotype and multipotential differentiation assay of DPCs. ((a)–(d)) DPCs exhibited a mesenchymal stem cell phenotype. Immunofluorescent staining demonstrated that DPCs expressed CD44 (a) and CD90 (b), but did not express CD31 (c). Flow cytometry analysis further demonstrated a high positive expression rate of CD90 and CD105, as well as a low positive expression rate of CD31 (d). ((e)–(h)) Multipotential differentiation capacity of DPCs. (e) Adipogenic differentiation of DPCs. The intracellular lipid droplets were formed after induction for 2 weeks and were detected by Oil-Red O staining. (f) Osteogenic differentiation of DPCs. Calcium nodules were formed after induction for 2 weeks and detected by Alizarin Red-S staining. (g) SMC differentiation of DPCs. A proportion of the DPCs were positive for *α*-SMA after induction for 1 week. (h) Neuronal differentiation of DPCs. A proportion of DPCs (white arrow) exhibited a neuron-like morphology and were positive for neuron-specific *β*3-Tubulin after induction for 1 week. Scale bar = 50 *μ*m ((a)–(c), (f)) and 20 *μ*m ((e), (g), (h)).

Immunofluorescent staining showed that DPCs were positive for the MSC markers CD44 (Figure 3(a)) and CD90 (Figure 3(b)), but were negative for the vascular endothelial cell marker CD31 (Figure 3(c)). Flow cytometry analysis further revealed the high positive expression rate of CD90 (99.78%) and CD105 (80.86%), and the low positive expression rate of CD31 (0.09%; Figure 3(d)). Based on the similar expression patterns of MSC markers with BMSCs (Supplementary Figures  3(a)–3(d)), we speculated that DPCs may be capable of multipotent differentiation, similar to BMSCs.

### 3.4. Multipotential Differentiation Assay

#### 3.4.1. Adipogenic Differentiation

After induction for 2 weeks, intracellular lipid droplets were formed and detected by Oil-Red O staining. The lipid droplets in DPCs were smaller and less obvious (Figure 3(e)) compared with those of BMSCs (Supplementary Figure  3(e)).

#### 3.4.2. Osteogenic Differentiation

After induction for 2 weeks, calcium nodules were formed and detected by Alizarin Red-S staining. The calcium nodules outside DPCs were not massively formed (Figure 3(f)), as compared with those outside BMSCs (Supplementary Figure  3(f)).

#### 3.4.3. SMC Differentiation

After culturing DPCs and BMSCs individually in SMC differentiation medium for 1 week, the expression of *α*-SMA, a known, typical SMC marker, was revealed with immunofluorescent staining (Figure 3(g); Supplementary Figure  3(g)). The positive expression ratio was 55.33 ± 14.76% and 68.67 ± 7.50% for DPCs and BMSCs, respectively. There was no significant difference between these expression levels (*P* > 0.05).

#### 3.4.4. Neuronal Differentiation

DPCs and BMSCs were cultured individually in neuronal differentiation medium for 1 week. We observed that some of these cells exhibited a neuron-like morphology and were stained positive for neuron-specific *β*3-Tubulin (Figure 3(h), Supplementary Figure  3(h)). The positive expression ratio for DPCs and BMSCs was 10.43 ± 4.76% and 10 ± 4.69%, respectively. However, there was no significant difference between these expression levels (*P* > 0.05).

Therefore, these results indicate that DPCs differentiated into adipocytes, osteoblasts, SMCs, and neurons under specific conditions, but the differentiation capacity of the mesenchymal lineages was weaker for DPCs than for BMSCs.

### 3.5. NTF Secretion from DPCs

**Figure 4:**
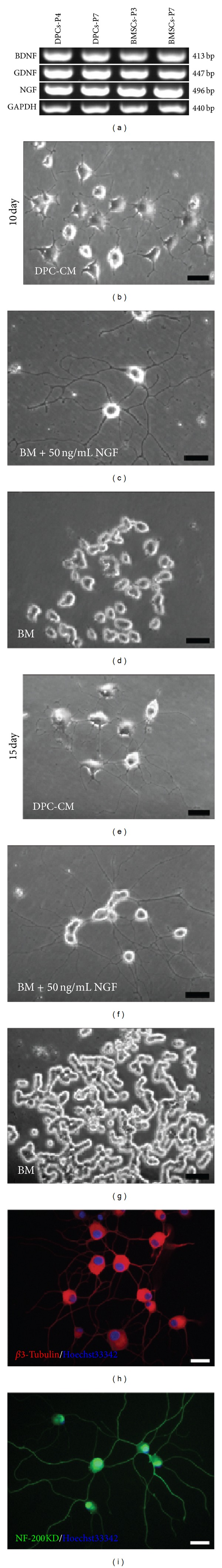
DPC-CM stimulated neuron-like differentiation of PC12 cells. (a) RT-PCR analysis revealed a similar expression for the NTFs NGF, GDNF, and BDNF in DPCs on passage 4 (DPCs-P4) and passage 7 (DPCs-P7), as well as BMSCs on passage 3 (BMSCs-P3) and passage 7 (BMSCs-P7). ((b)–(i)) Neuronal differentiation of PC12 cells. After culturing in DPC-CM for 10 days, PC12 cells grew several neurites from their somas (b), though the lengths of the neurites were shorter than those for positive control groups cultured in BM supplemented with 50 ng/mL NGF (c), but were significantly longer than those for the negative control groups cultured in BM (d). After culturing for 15 days, the neurites of PC12 cells cultured in DPC-CM increased in length (e). The neurites of the positive control groups interlaced to form a net (f). By contrast, the negative control groups proliferated but bore no neurites (g). Immunofluorescent cytochemical staining demonstrated the neuronal differentiation of PC12 cells that expressed *β*3-Tubulin (h) and NF-200KD (i). Scale bar = 40 *μ*m ((b)–(g)) and 20 *μ*m ((h), (i)).

We examined whether DPCs could secrete NTFs, and specifically NGF, GDNF, and BDNF. We found that these factors demonstrate similar gene expression patterns in DPCs and BMSCs at the mRNA level (Figure 4(a)). Moreover, we detected NGF expression in DPCs and BMSCs using a western blot analysis, and that NGF expression was higher in DPCs than BMSCs (Supplementary Figures  4(a) and 4(b)). Because each of these NTFs participate in the differentiation of neural precursor cells into neurons, we examined their morphological changes after PC12 cells were cultured in BM supplemented with 50 ng/mL NGF, DPC-CM, and BM. After culturing in DPC-CM for 10 days, PC12 cells exhibited several neurites growing from their somas (Figure 4(b)). The lengths of these neurites were shorter than those for the positive control groups, which were cultured in BM supplemented with 50 ng/mL NGF (Figure 4(c)). However, the neurites of PC12 cells cultured in DPC-CM were significantly longer than those for the negative control groups that were cultured in BM (Figure 4(d)). After culturing for 15 days in DPC-CM, the neurite lengths of PC12 cells increased significantly (Figure 4(e)). The positive control groups showed an evident neurite extension, and the majority of the differentiated cells appeared mostly rounded and interconnected by their neurites (Figure 4(f)). In contrast, the PC12 cells of the negative control groups actively proliferated, but did not show neurite outgrowth (Figure 4(g)). Additionally, immunofluorescent staining demonstrated the neuronal differentiation of PC12 cells with expression of *β*3-Tubulin (Figure 4(h)) and NF-200KD (Figure 4(i)). Interestingly, we observed that BMSC-CM only stimulated proliferation of PC12 cells, and did not promote neural differentiation (Supplementary Figure  5(a)) when compared with DPC-CM (Supplementary Figure  5(b)).

**Figure 5:**
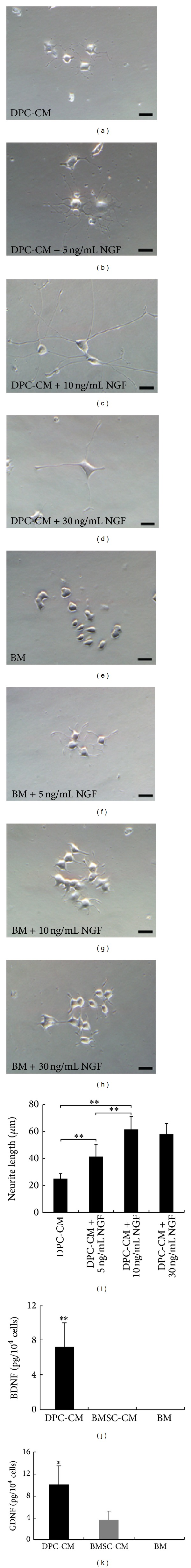
DPCs promoted neurite outgrowth in PC12 cells and secreted BDNF and GDNF. ((a)–(h)) After culturing for 4 days, the average neurite lengths of PC12 cells cultured in DPC-CM supplemented with various concentrations of NGF ((a)–(d)) were longer than those of cells cultured in BM supplemented with the same concentrations of NGF ((e)–(h)), as indicated by phase contrast microscopic observation. (i) The PC12 cells cultured in DPC-CM supplemented with 10 ng/mL NGF displayed the longest average neurite lengths compared with other concentrations of NGF (0 ng/mL, 5 ng/mL, and 30 ng/mL), but there was no statistical difference with high dose NGF (30 ng/mL) (*P* > 0.05). ((j), (k)) The amount of BDNF (j) and GDNF (k) secreted per 10 thousand cells in 5 mL DPC-CM, BMSC-CM, and BM for 24 h. Scale bar = 20 *μ*m ((a)–(h)). *P < 0.05. **P < 0.01.

Although DPC-CM stimulated neuronal differentiation of PC12 cells, their neurite lengths were lesser than those for cells in the positive control groups. We considered the possibility that the NGF concentration for DPC-CM was insufficient for promoting or activating neurite elongation. Consequently, PC12 cells were cultured in DPC-CM or BM supplemented with various concentrations of NGF (0, 5, 10, and 30 ng/mL). After culturing for 4 days, we found that the average neurite length of PC12 cells cultured in DPC-CM supplemented with NGF was longer than that for cells cultured in BM supplemented with the same NGF concentrations (Figures 5(a)–5(h)). Among them, the PC12 cells cultured in DPC-CM supplemented with 10 ng/mL NGF displayed the longest neurite extensions (Figure 5(i)). These results indicate that NGF stimulated neuronal differentiation of PC12 cells, and the significant neurite elongation required a high dose of NGF (>10 ng/mL) in DPC-CM. Other NTFs were present in the DPC-CM, which had synergistic functions with NGF that promoted neurite outgrowth.

Finally, using ELISA assay, we detected secretion of NGF, BDNF, and GDNF in DPC-CM. Unexpectedly, we found that the NGF levels in DPC-CM were undetectable (data not shown here). In contrast, the amounts of BDNF and GDNF secreted per ten thousand cells in DPC-CM for 24 h were significantly higher than that in BMSC-CM (BDNF, 7.28 ± 2.75 pg/10^4^ cells versus none detected; GDNF, 10.14 ± 3.40 pg/10^4^ cells versus 3.75 ± 1.58 pg/10^4^ cells; Figures 5(j) and 5(k)). Thus, we considered that in DPC-CM, at least BDNF and GDNF, but not NGF, were crucial NTFs that played important roles in stimulating neuronal differentiation of PC12 cells.

### 3.6. Isolation and Characterization of Neural Crest Stem Cell-Like Cells

**Figure 6:**
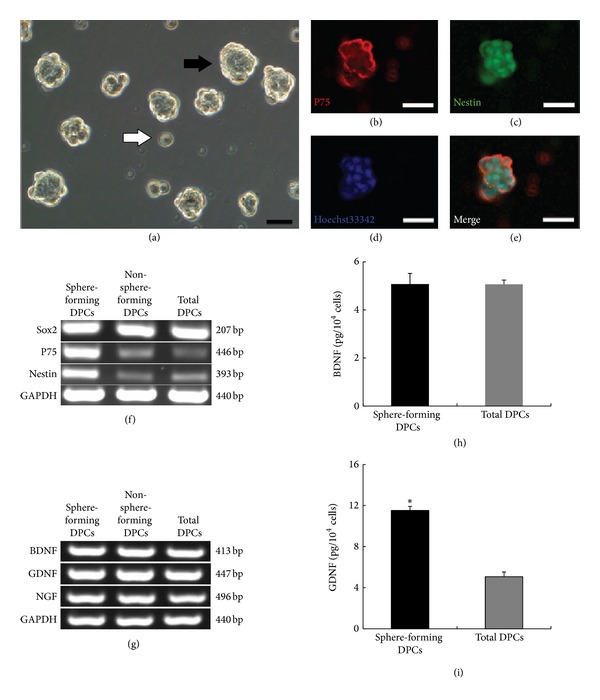
Isolation and characterization of NCSCs-like cells. (a) After suspension in culture for 7 days, neurosphere-like spheres were generated from DPCs (black arrow), along with non-sphere-forming DPCs (white arrow). ((b)–(e)) Coexpression of Nestin (b) and P75 (c) in spheres were detected by double immunostaining. Nuclei were stained with Hoechst 33342 (d). The images were merged (e). (f) RT-PCR showed a higher expression level of NCSCs-specific markers in sphere-forming DPCs compared with non-sphere-forming DPCs and total DPCs on the same passage. (g) RT-PCR revealed similar expression levels of NTFs in sphere-forming DPCs, non-sphere-forming DPCs, and total DPCs. ((h), (i)) The amount of BDNF (h) and GDNF (i) secreted per 10 thousand sphere-forming DPCs and total DPCs in 5 mL supernatant for 24 h. Scale bar = 20 *μ*m ((a), (b)–(e)). *P < 0.05.

According to previous reports, the hair follicle papilla is a primary source of skin-derived precursors (SKPs), and SKPs have NCSCs-like properties of forming neurospheres under serum-free culture conditions containing N-2, B-27, bFGF, and EGF. We suspected that within the monolayer-expanded DPCs, the neurosphere-forming cells were major producers of BDNF and GDNF. To confirm our hypothesis, DPCs were cultured in suspension at low density in serum-free sphere-forming medium. After 7 days, neurosphere-like spheres were generated (Figure 6(a)), which were identified with double immunostaining by coexpression of the NCSCs markers P75 and Nestin (Figures 6(b)–6(e)). The percentage of sphere-forming DPCs was 1.14 ± 0.03%. Under adherent culture conditions, the morphology of sphere-forming DPCs and non-sphere-forming DPCs assumed clonal growth and dispersed elongated spindle cells, respectively. RT-PCR analysis revealed that, compared with total DPCs and non-sphere-forming DPCs, sphere-forming DPCs showed higher levels of Nestin and P75 expression (Figure 6(f)). These results imply that sphere-forming DPCs likely have more robust differentiation potential than total DPCs. At the mRNA level, we found that the sphere-forming DPCs expressed NTFs at levels similar to those of other cells (Figure 6(g)). In contrast, by ELISA analysis, similar amounts of BDNF (5.07 ± 0.45 pg/10^4^ cells versus 5.06 ± 0.18 pg/10^4^ cells) and a more abundant amount of GDNF (11.52 ± 0.40 pg/10^4^ cells versus 5.07 ± 0.45 pg/10^4^ cells) were detected in the supernatants from monolayer cultured sphere-forming DPCs compared with total DPCs (Figures 6(h) and 6(i)). These results demonstrated that sphere-forming DPCs might be a major subpopulation of cells for secreting GDNF.

## 4. Discussion

In this study, we confirmed that the monolayer-expanded DPCs had differentiation potentials similar to those of BMSCs, but DPCs' capacities for mesodermal lineage differentiation were weaker than those for BMSCs. Intriguingly, the DPCs released greater quantities of BDNF and GDNF into the extracellular environment than did BMSCs, and DPC-CM significantly stimulated neuronal differentiation of PC12 cells. Unlike BMSCs, we found that DPCs did not secrete detectable levels of NGF. Furthermore, we observed that within the monolayer-expanded cells, the sphere-forming DPCs were a major subpopulation of cells that released GDNF.

DPCs are specialized mesenchymal cells, which are located inside the hair bulb and are required for regulating epithelial stem cells during hair morphogenesis, hair induction, and hair cycle regulation [22]. The hair dermal papilla is also considered a reservoir of multipotent stem cells [23]. In line with other reports [23, 24], we observed that DPCs from the rat whisker follicle have the capacity to differentiate into adipocytes, osteoblasts, SMCs, and neurons. However, we found that DPCs had a weaker capacity for differentiating into mesenchymal lineages. The transcription factor Sox2, which is a marker for DPCs during anagen, can regulate the self-renewal of stem cells and maintain the stemness [23, 25, 26]. Based on our results, we believe that the expression level of Sox2 is not related to the differentiation capacity of the mesenchymal lineages. Sox2 is highly expressed in neural stem cells and epithelia stem cells [27, 28]. Therefore, we speculate that within hair papilla, Sox2 negatively regulated differentiation of DPCs toward mesenchymal derivatives. On the other hand, Sox2 may function in hair follicle morphogenesis, controlling DPCs differentiation into neural cells more easily, and producing higher levels of NTFs, including BDNF and GDNF.

Similar to BMSCs, craniofacial hair follicle papilla cells are considered mesenchymal cells, but they originate from the neural crest [13, 14]. Jinno et al. [14] reported that in craniofacial skin, DPCs are a primary source of SKPs, which have NCSCs-like properties. In here, the differentiation potential combined with the expression of typical NCSCs markers indicates that DPCs have certain properties that are similar to those of NCSCs, but they are distinct from the original NCSCs. The transcription factor Sox10 is a hallmark of NCSCs and is persistently expressed after they differentiate into Schwann cells and melanocytes, but disappears after they differentiate into neurons [29–31]. In this study, we found that under Schwann cell-inducing conditions, which included addition of forskolin and neuregulin-*β*1, DPCs failed to express the Schwann cells markers Sox10, S100-*β*1, and GFAP (data not shown here). These results are different from those presented in other reports [32]. We suggest that the cell cultivation method and the donor ages eventually influenced the cellular differentiation capacity. In our work, DPCs were expanded in DMEM/F-12 media comprising 10% FBS and 10 ng/mL bFGF, and not under neurosphere culture conditions. Under this more complex monolayer culture condition, we found that DPCs gradually lost the capacity to differentiate into Schwann cells and melanocytes, but kept the potential to differentiate into neurons and mesenchymal lineage-like osteocytes and adipocytes. Additionally, unlike other studies, our DPCs were isolated from adult rat whisker papilla and not from newborn rats [14]. The differentiation potential of DPCs into Schwann cells or melanocytes is possibly limited to the embryonic or neonatal stages of rats and is lost with age.

NTFs such as NGF, BDNF, and GDNF are essential for neuronal survival and functions. BDNF not only stimulates neurite outgrowth for several neuronal cell types* in vitro*, but also stimulates regrowth of multiple descending axon tracts within the spinal cord following injury [33–35]. In addition, transplantation of neural stem cells overexpressing GDNF enhanced neurogenesis in rats after stroke [36]. However, to date, we are unaware of reports concerning the neurotrophic secretion characteristics of DPCs. BMSCs also produce NTFs such as NGF, BDNF, and VEGF [37]. However, we observed that PC12 cells did not exhibit neuronal differentiation in BMSC-CM and that BDNF and GDNF concentrations in BMSC-CM were significantly lower than in DPC-CM. Moreover, we observed that, compared with non-sphere-forming DPCs, sphere-forming DPCs secreted greater amounts of GDNF. Therefore, we speculate that compared to BMSCs, the multipotent DPCs, especially sphere-forming DPCs, may better protect neurons from death and promote neural regeneration following transplantation into areas lesioned by stroke or spinal cord injury. To examine this possibility, we intend to directly transplant DPCs, sphere-forming DPCs, and BMSCs from the same donor into the damaged brain of rats subjected to ischemic strokes, and further compare the therapeutic effects of these cell types.

## 5. Conclusion

In summary, our data demonstrate that DPCs from craniofacial hair follicle dermal papilla have adult stem cell properties and a greater capacity to secrete BDNF and GDNF than do BMSCs. We suggest these cells are a promising source for autologous cell therapy in treatments for CNS injury.

## Supplementary Material

We described the method of isolating and culturing BMSCs, and we further analyzed the phenotype and the multipotential differentiation capacity of BMSCs. Moreover, we detected NGF expression in DPCs and BMSCs using a western blot analysis and examined the morphological changes of PC12 cells after culturing in BMSC-CM.

## Figures and Tables

**Table 1:** List of all primers used with PCR and amplification conditions.

Gene	Primer sequence	Annealing temperature
ALP	TCCATGGTGGATTATGCTCA TTCTGTTCCTGCTCGAGGTT	60°C

Nestin	ACTGCAGAAGAGGACCTGGA AGTTCCCACTCCTGTGGTTG	56°C

Sox2	GCACATGAACGGCTGGAGCAACG TGCTGCGAGTAGGACATGCTGTAGG	65°C

P75	GGCTACTACCAGGACGAGGAG GCCAAGATGGAGCAATAGACA	58°C

Sox9	GTGCTGAAGGGCTACGACTGGA GTTGTGCAGATGCGGGTACTGG	64°C

Twist1	CATCCTTGTGGACTTTCTCCA GCATTTTACCATGGGTCATCA	60°C

AP2*α*	TGGGCACTGTAGGTCAATCTC GTTAATAGGGATGGCGGAGAC	60°C

BDNF	TGGCTGACACTTTTGAGCAC GCAGCCTTCCTTCGTGTAAC	56°C

GDNF	GACTCCAATATGCCCGAAGA ATGGTAAACCAGGCTGTCGT	56°C

NGF	GGACGCAGCTTTCTATCCTG GTCCGTGGCTGTGGTCTTAT	56°C

GAPDH	ATGGGAAGCTGGTCATCAAC GGATGCAGGGATGATGTTCT	58°C

**Table 2:** Detailed information concerning the primary antibodies.

Name of antibody	Antibody dilution	Company	Catalogue number
P75	1 : 400	Millipore	#AB1554
Sox10	1 : 100	R&D	MAB2864
Nestin	1 : 50	eBioscience	14-5843
Sox9	1 : 500	Millipore	AB5535
CD44	1 : 800	Cell signal	#5640
CD90	1 : 50	Millipore	MAB1406
CD105	1 : 100	Millipore	#05-1424
CD31	1 : 20	Abcam	ab28364
*α*-SMA	1 : 200	Abcam	ab3280
*β*3-Tubulin	1 : 200	Abcam	ab18207
NF-200KD	1 : 200	Millipore	MAB5262
NGF	1 : 1000	Abcam	ab6199
HNK1	1 : 100	Boshide	BM0325

## Abbreviations

ALP: Alkaline phosphatase
BDNF: Brain-derived neurotrophic factor
BM: Basal medium
BMSC-CM: Bone marrow mesenchymal stem cell-conditioned medium
BMSCs: Bone marrow mesenchymal stem cells
CNS: Central nervous system
DPC-CM: Dermal papilla cell-conditioned medium
DPCs: Dermal papilla cells
EGF: Epidermal growth factor
FBS: Fetal bovine serum
GAPDH: Glyceraldehyde-3-phosphate dehydrogenase
GDNF: Glial cell line-derived neurotrophic factor
MSC: Mesenchymal stem cell
NCSCs: Neural crest stem cells
NGF: Nerve growth factor
NTFs: Neurotrophic factors
PBS: Phosphate buffered saline
PC12 cells: Rat pheochromocytoma cell line
SKPs: Skin-derived precursors
SMC: Smooth muscle cell.
