# Supplementary material for: Multipotent Neural Crest Stem Cell-Like Cells from Rat Vibrissa Dermal Papilla Induce Neuronal Differentiation of PC12 Cells
Source: Biomed Res Int. 2014 Jun 18;2014:186239. doi: 10.1155/2014/186239 (PMC4086521; doi:10.1155/2014/186239)
Supplement: Supplementary file 1 — We described the method of isolating and culturing BMSCs, and we further analyzed the phenotype and the multipotential differentiation capacity of BMSCs. Moreover, we detected NGF expression in DPCs and BMSCs using a western blot analysis and examined the morphological changes of PC12 cells after culturing in BMSC-CM. [file 186239.f1.pdf]

Supplementary materials

Materials and methods

Isolation and cultivation of BMSCs

For BMSCs isolation, the hind limbs of Wistar rats (200–250 g, n = 7) were dissected and maintained in PBS containing 5% (v/v) penicillin and streptomycin under sterile conditions. After removal of the musculature and connective tissue, the femur and tibia were rinsed with PBS, and their epiphyses were carefully cut with a bone cutter. The bone marrow was flushed using a 10 mL syringe containing PBS and then centrifuged for 5 min at 1,500 rpm at room temperature. The pellet was suspended in PBS, filtered through 40-μm filters, and then re-centrifuged at 1,500 rpm for 5 min. Next, the pellet was suspended in 10 mL proliferation medium and cultured in a 100-mm culture dish. Half of the medium was changed every 3 days. The cells were passaged when they reached ~90% confluence.

Western blot

Proteins (20 μg/ lane) were fractionated by SDS-PAGE and electrotransferred to a polyvinylidene difluoride membrane. These blots were first incubated for 1 h in a blocking buffer consisting of 0.1% Tween-20 (Sigma) and 5% nonfat powdered milk, and then incubated with primary antibody against NGF (Abcam, Cambridge, UK) at 4◦C overnight. A horseradish peroxidase-conjugated goat anti-rabbit secondary antibody (Proteintech, Chicago, USA) was used, and antigen-antibody complexes were detected by chemiluminescence using the BeyoECL Plus kit (Bi Yuntian, Shanghai, China).

Supplementary Figure 1: Isolation and cultivation of BMSCs. (a) After culture for 1 week, the spindled-shaped adherent cells were observed and formed cell colonies. (b) BMSCs at passage 2 (BMSCs-P2) showed a typical fibroblast-like morphology and were arranged in swirl. (c) BMSCs at passage 7 (BMSCs-P7) partially changed to a flattened and spread out morphology. Scale bar = 200 μm ((a), (c)), 100 μm (b).

Supplementary Figure 2: Identification of neural crest-derived DPCs. ((a), (b)) Immunofluorescent cytochemical staining revealed expression of NCSCs-specific markers in DPCs. After passage, they were negative for Sox10 (a), but were positive for P75 (a) and Nestin (b). Nuclei were stained with Hoechst 33342. Scale bar = 100 μm (a), 50 μm (b).

Supplementary Figure 3: Mesenchymal stem cell phenotype and multipotential differentiation assay of BMSCs. ((a)–(d)) BMSCs exhibited a mesenchymal stem cell phenotype. Immunofluorescent staining demonstrated that BMSCs expressed CD44 (a), CD90 (b), did not express CD31 (c). Flow cytometry analysis further demonstrated a high positive expression rate of CD44 and CD90, as well as a low positive expression rate of CD31 (d). ((e)–(h)) Multipotential differentiation capacity of BMSCs. (e) Adipogenic differentiation of BMSCs. The intracellular lipid droplets were formed after induction for 2 weeks and were detected by Oil-Red O staining. (f) Osteogenic differentiation of BMSCs. Calcium nodules were formed after induction for 2 weeks and detected by Alizarin Red-S staining. (g) SMC differentiation of BMSCs. A proportion of the BMSCs were positive for α-SMA after induction for 1 week. (h) Neuronal differentiation of BMSCs. A proportion of BMSCs (white arrow)

exhibited a neuron-like morphology and were positive for neuron-specific β3-tubulin after induction for 1 week. Scale bar = 50 μm ((a)–(c), (f)), 20 μm ((e), (g), (h)).

Supplementary Figure 4: Western blot revealed expression of NGF in DPCs. (a) By western blot analysis, a higher expression level of NGF was detected in DPCs compared with BMSCs. (b) The relative expression to GAPDH was 0.41 ± 0.07 (DPCs) and 0.26 ± 0.06 (BMSCs), respectively. *$P < 0.05$.

Supplementary Figure 5: BMSC-CM stimulated proliferation of PC12 cells. ((a), (b)) Phase contrast images of PC12 cells cultured for 15 days in BMSC-CM (a) and DPC-CM (b). PC12 cells proliferated actively in BMSCs-CM (a), by contrast, PC12 cells morphologically differentiated into neuron-like cells in DPCs-CM (b). Scale bar=20μm.

Supplementary Figure 1

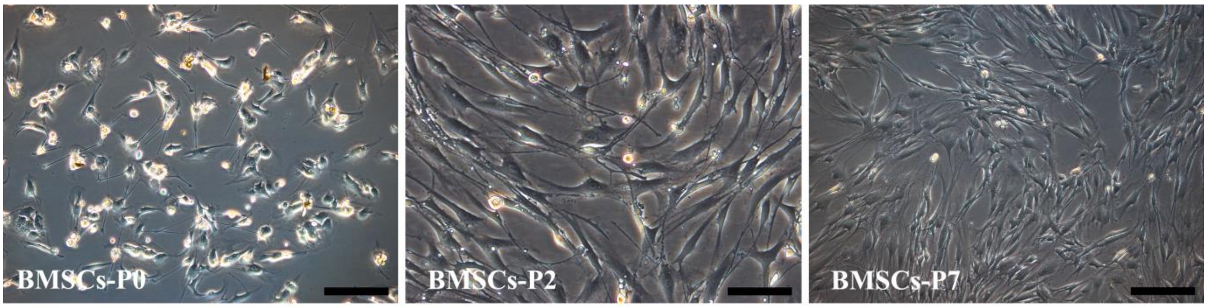

(a) (b) (c)

Supplementary Figure 2

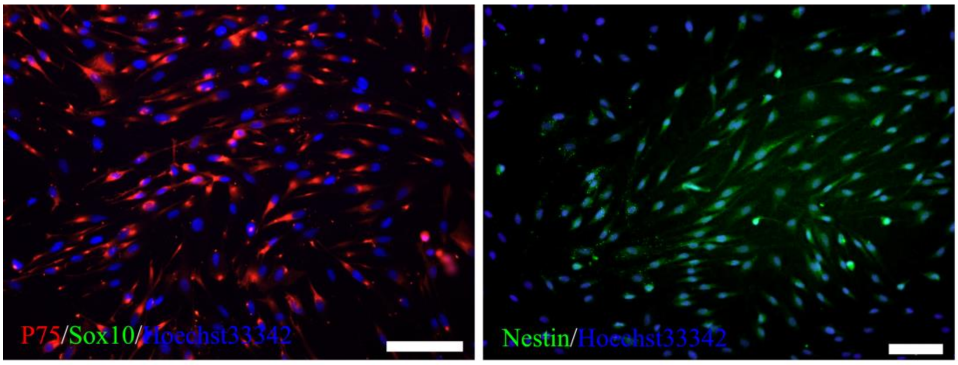

(a) (b)

Supplementary Figure 3

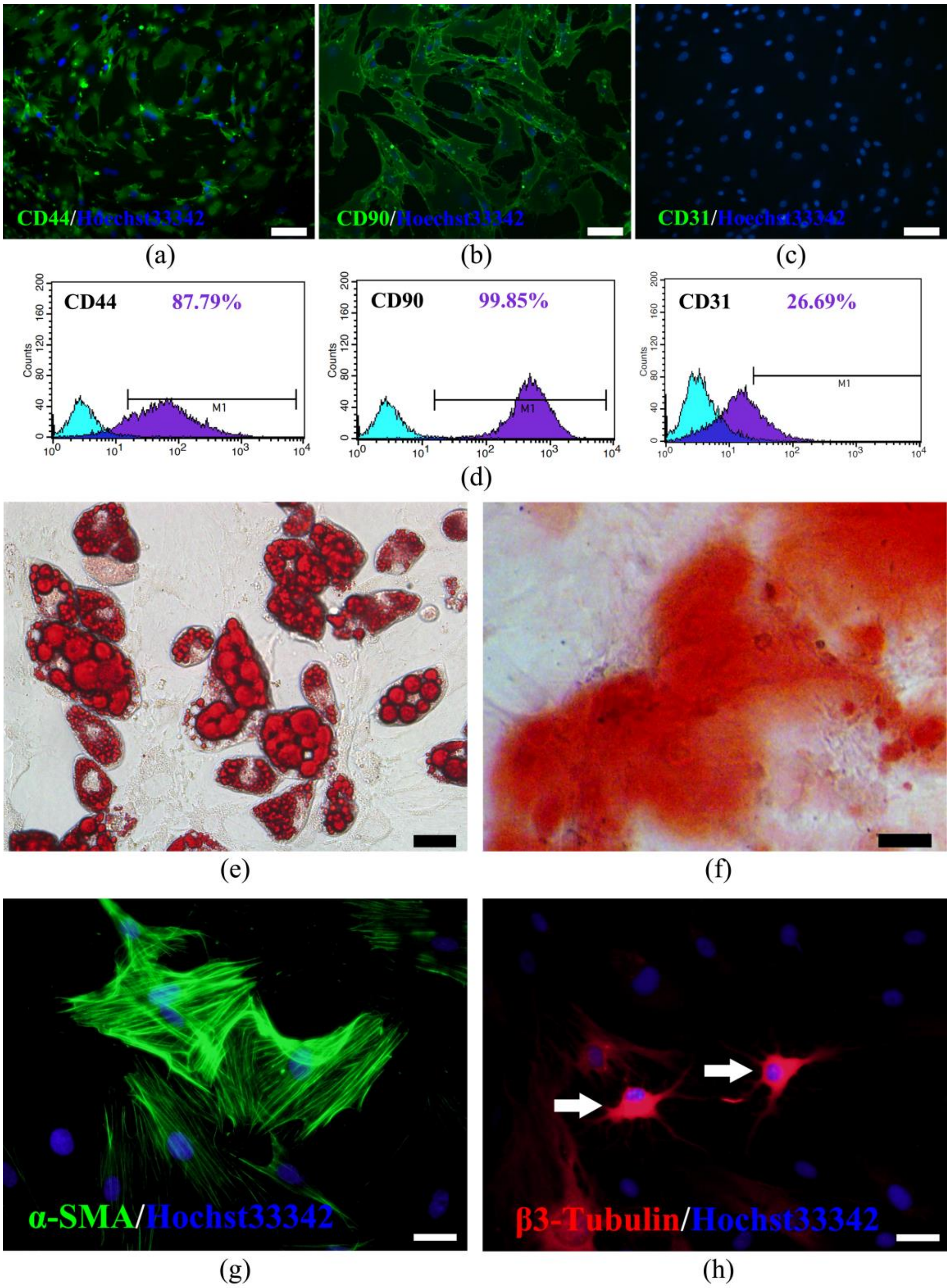

Supplementary Figure 4

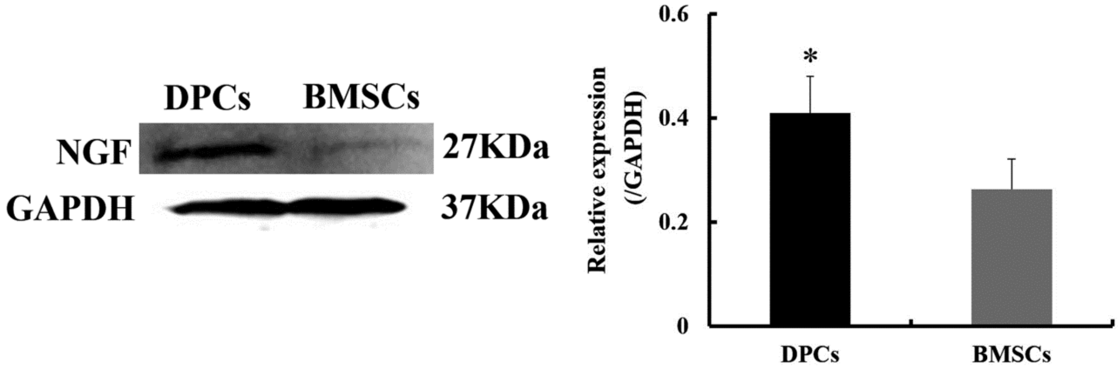

(a) (b)

Supplementary Figure 5

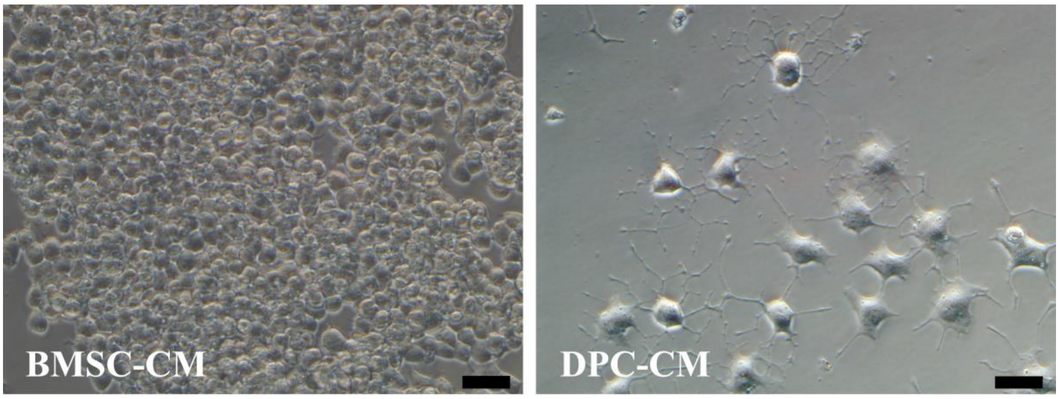

(a) (b)
